# Machine learning methods to predict mechanical ventilation and mortality in patients with COVID-19

**DOI:** 10.1371/journal.pone.0249285

**Published:** 2021-04-01

**Authors:** Limin Yu, Alexandra Halalau, Bhavinkumar Dalal, Amr E. Abbas, Felicia Ivascu, Mitual Amin, Girish B. Nair

**Affiliations:** 1 Department of Pathology, Beaumont Health System, Royal Oak, MI, United States of America; 2 Department of Internal Medicine, Beaumont Health System, Royal Oak, MI, United States of America; 3 Division of Pulmonary and Critical Care Medicine, Beaumont Health System, Royal Oak, MI, United States of America; 4 Department of Cardiovascular Medicine, Beaumont Health System, Royal Oak, MI, United States of America; 5 Department of General Surgery, Beaumont Health System, Royal Oak, MI, United States of America; Ohio State University Wexner Medical Center Department of Surgery, UNITED STATES

## Abstract

**Background:**

The Coronavirus disease 2019 (COVID-19) pandemic has affected millions of people across the globe. It is associated with a high mortality rate and has created a global crisis by straining medical resources worldwide.

**Objectives:**

To develop and validate machine-learning models for prediction of mechanical ventilation (MV) for patients presenting to emergency room and for prediction of in-hospital mortality once a patient is admitted.

**Methods:**

Two cohorts were used for the two different aims. 1980 COVID-19 patients were enrolled for the aim of prediction ofMV. 1036 patients’ data, including demographics, past smoking and drinking history, past medical history and vital signs at emergency room (ER), laboratory values, and treatments were collected for training and 674 patients were enrolled for validation using XGBoost algorithm. For the second aim to predict in-hospital mortality, 3491 hospitalized patients via ER were enrolled. CatBoost, a new gradient-boosting algorithm was applied for training and validation of the cohort.

**Results:**

Older age, higher temperature, increased respiratory rate (RR) and a lower oxygen saturation (SpO2) from the first set of vital signs were associated with an increased risk of MV amongst the 1980 patients in the ER. The model had a high accuracy of 86.2% and a negative predictive value (NPV) of 87.8%. While, patients who required MV, had a higher RR, Body mass index (BMI) and longer length of stay in the hospital were the major features associated with in-hospital mortality. The second model had a high accuracy of 80% with NPV of 81.6%.

**Conclusion:**

Machine learning models using XGBoost and catBoost algorithms can predict need for mechanical ventilation and mortality with a very high accuracy in COVID-19 patients.

## Introduction

The number of infections related to the severe acute respiratory syndrome coronavirus-2 (SARS-CoV-2) and causing coronavirus disease 2019 (COVID-19) has increased exponentially with over 4 million cases reported in the US alone. Many states and hospital systems have experienced considerable challenges with the unexpected number of cases with a strain on an already fragile health system, causing multiple hospitals to reach or exceed capacity [1]. The majority of patients experience mild disease but approximately 15% -20% of symptomatic patients progress to severe pneumonia requiring hospitalization [2]. Current evidence suggests important derangements within the immune system and the coagulation cascade in COVID-19 patients [3, 4].

Observational studies have shown several features, associated with increased risk of hospitalization in COVID-19 including older age, male sex, obesity, admission oxygen saturation (SpO2) less than 88%, respiratory rate greater than 24/minute, comorbid conditions such as diabetes, hypertension, chronic kidney disease and lab values like, elevated troponin level, C reactive protein level > 200 and D-dimer level > 2500 [5–7]. All these studies also point to a high mortality in intubated patients over 50%. Other reports suggest that patients over the age of 65, and those with co-morbid conditions are at a higher risk of mortality, ranging from 4.3%–7.5% [8–10].

There is an urgent need for disease stratification during the pandemic and several statistical models are being developed based on observational studies. However, despite various retrospective associations, it is still unclear if an individual patient in the emergency room (ER) with mild to moderate disease is at risk of progression to severe disease. Machine learning (ML) algorithms are designed to scrutinize big data from both structured and unstructured data and gather information without bias. Real time efficient management of patient and hospital resource allocation would require development of a predictive model, which can accurately classify COVID-19 patients at risk of invasive mechanical ventilation (MV) and death.

We hypothesized a parsimonious model with fewer parameters including vital signs and demographics at the time of presentation would be helpful in the ER for determining need for intubation and mechanical ventilation, while a complicated profile with laboratory values will be beneficial for hospitalized patients to prognosticate mortality.

## Methods

This is a retrospective multicenter study of all patients with COVID-19 patients, who presented to the ER at Beaumont Health, Michigan’s largest health care system, consisting of eight hospitals. We developed two separate decision-tree-based, ensemble ML algorithm with two aims of study: 1. prediction of MV, and 2. Mortality. This study was approved by Beaumont Health Institutional Review Board 2020–125 and all data were fully anonymized. The patients’ COVID-19 infection was confirmed by a positive SARS-CoV-2 nucleic acid by real-time fluorescent RT-PCR test of respiratory tract or blood specimens.

### Participates

- Prediction of MV: A total of 1,980 COVID-19 patients who were evaluated at ER between 2/20/2020 and 5/5/2020 were enrolled. The patients who visited an ER department between 2/20/2020 and 4/17/2020 were used as the training and testing cohort. COVID-19 patients who visited ER between April 18 and May 5, 2020 were enrolled as the prospective validation cohort.
- Prediction of Mortality. A total of 3,491 hospitalized COVID-19 in-patients were enrolled. They visited ER departments of Beaumont Health and were subsequently admitted between 2/1/2020 and 5/4/2020. Survivors were hospitalized for 8.4 days on average; demised patients were hospitalized for 11.1 days on average. More clinical and laboratory data were collected on these patients.

### Data collection

Information including demographics, past smoking and drinking history, past medical history and vital signs at ER, laboratory values, and treatments were used as independent features of the prediction models. They were collected from EPIC EMR system at Beaumont Health using Structured Query Language (SQL) queries.

### Data cleaning

Prediction of MV: Same technique of data cleaning was applied to both the cohort between 2/20/2020 and 4/17/2020 and the cohort between 4/18/2020 and 5/5/2020. Missing information of smoking and alcohol information was replaced with “Not Asked”. Subsequently the categorical values of smoking and alcohol history were converted into ordinal discrete values as follows: ‘Never’, 0; ’Quit’, 1; ’Not Asked’, 2, Passive’, 3, ’Yes’, 4. Missing value of alcohol history was substituted by “Not Asked”. The categorical values of alcohol history were changed into ordinal discreate values as follows: ’No’, 0; ’Never’, 0, ‘Not Currently’, 1; ’Not Asked’, 2, ’Yes’, 3. The missing values of BMI, oxygen saturation (spO2), systolic blood pressure (sBP), diastolic blood pressure (dBP), pulse, RR, and temperature were replaced by their corresponding mean values.

Prediction of Mortality: 34 clinically important features having valid values in more than 3,000 out of 3,491 patients were selected. They included demographic information: Age, Sex, Race, BMI; past history of hypertension (HTN), hyperlipidemia (HLD), diabetes mellitus (DM), cardiovascular disease (CAD), heart failure (HF), peripheral artery disease (PAD), atrial fibrillation (AF), cerebrovascular accident and transient ischemic attack (CVA/ITA), venous thromboembolism (VTE), pulmonary hypertension (PH), chronic respiratory failure, chronic lung disease, chronic kidney disease (CKD), GI bleeding, Immunocompromised/suppressed, connective tissue disease/autoimmune disease (CTD/AI disease), malignancy (Cancer); vital signs taken at ER: temperature (Temp), systolic blood pressure (sBP), diastolic blood pressure (dBP), pulse, respiration rate; course and treatment: length of stay in days (LOS), therapeutic anticoagulation (tAC), therapeutic anticoagulation duration in days (tAC_dur), Steroids, steroid treatment duration in days (steroid_dur), mechanical ventilation (vented), need for vasopressors (pressors), need for ICU admission (ICU_adm). Missing values of numerical features, including BMI, spO2, sBP, dBP, pulse, respiration rate, and temperature, were replaced by their corresponding mean values. Categorical features are not encoded because of CatBoost’s default ability handling categorical features.

### Statistic

In baseline characteristics, continuous features are presented as means with standard deviations (SDs), and comparisons between groups were analyzed by performing two-sided Student’s t-test. Categorical variables were represented as frequencies and percentages and they were compared using Chi-square test (if cell counts equal to or more than 5) or Fisher’s exact test (if cell counts below 5). Statistics analysis was conducted by SAS (version 9.4, SAS Institute, Cary, NC).

### Machine leaning algorithms

For prediction of mechanical ventilation, we implemented a classification algorithm based on XGBoost (https://github.com/dmlc/xgboost/). Designed for speed and performance, XGBoost is decision-tree-based ensemble Machine Learning algorithm [11]. It uses an ensemble method that fits each iteration of the new model with residuals from previous prediction in both regression and classification trees. Since its introduction in 2016, it has been credited for winning numerous data science competitions and improving industry applications [12]. We utilized k-fold cross-validation during training and hyperparameter optimization to prevent overfitting. Prediction of mortality was performed using CatBoost (https://catboost.ai/), a new gradient-boosting algorithm. It manages categorical features out-of-box and outperforms state-of-the-art machine-learning algorithms on popular publicly available data sets. In implementation, categorical features were indicated explicitly and CatBoost encodes them one-hot encoding.

Accuracy and AUC (Area Under the Curve) ROC (Receiver Operating Characteristics) curve were used to evaluate the performance of prediction models. Our algorithms were developed in Python (3.6.3) for data collection, data cleaning, feature engineering, machine learning training and testing. The development environments included PyCharm and Jupyter Notebook. The key libraries included Numpy, Pandas, Sklearn, Scipy, XGBoost, catBoost, imLearn, and matplotlib. The last decade has witnessed the rapid progress in machine learning and AI. Their adoption in medicine lags behinds other industries. Unexplainability is one of the major criticisms. In this study, we attempted to shed light on ML models in predicting COVID-19 patients’ clinical outcome using SHAP (SHapley Additive exPlanations). SHAP is a game theoretic approach to explain the output of any machine learning model [13]. It connects optimal credit allocation with local explanations using the classic Shapley values from game theory and their related extensions. SHAP values are the average of the marginal contributions across all permutations, providing global view of feature ranking and individual force view.

## Results

### Prediction of mechanical ventilation

A total of 1,980 unique patients were analyzed (Table 1). The average age was 63.2 ± 17.1 years old and 1,013 (51.2%) were male. 1,306 patients visited an ER department in Beaumont Health system between 2/20/2020 and 4/17/2020 and 674 of them between 4/18/2020 and 5/6/2020 for a COVID-19 related symptom. There are significant statistical differences in sex, race, BMI, smoking history, history of DM, lung disease and heart disease between those who were mechanically ventilated and those who were not.

**Table 1:** Clinical characteristics of patients in prediction of mechanical ventilation.

	Total Patients (n = 1,980)	No Mechanical Ventilation (n = 1,649)	Mechanical Ventilation (n = 331)	Significance
**Age**	63.2 ± 17.1	63.2 ± 17.6	63.2 ± 15.0	0.969
**Sex**	Male: 1,013 (51.2%)	Male: 813 (49.3%)	Male: 200 (60.4%)	0.001
	Female: 967 (48.8%)	Female: 836 (50.7%)	Female: 131 (39.6%)	
**Race**	Asian 32 (1.6%)	Asian 28 (1.7%)	Asian 4 (1.2%)	0.023
	AA: 1,117 (56.4%)	AA: 930 (56.5%)	AA: 187 (56.8%)	
	Caucasian: 725 (36.6%)	Caucasian: 615 (37.4%)	Caucasian: 110 (33.4%)	
	Other: 101 (5.1%)	Other: 73 (4.4%)	Other: 28 (8.5%)	
**BMI**	32.0 ± 9.0	31.7 ± 8.8	33.9 ± 10.0	0.001
**Smoking**	Never: 974 (49.2%)	Never: 830 (50.3%)	Never: 144 (43.5%)	0.002
	Not Asked: 412 (20.8%)	Not Asked: 346 (21.0%)	Not Asked: 66 (19.9%)	
	Passive: 5 (0.3%)	Passive: 3 (0.02%)	Passive: 2 (0.6%)	
	Quit: 507 (25.6%)	Quit: 399 (24.2%)	Quit: 108 (32.6%)	
	Yes: 82 (4.1%)	Yes: 71 (41.3%)	Yes: 11 (3.3%)	
**Alcohol**	Never: 97 (4.9%)	Never: 82 (5.0%)	Never: 15 (4.5%)	0.934
	No: 787 (39.7%)	No: 651 (39.5)	No: 136 (41.1%)	
	Not Asked: 471 (23.8%)	Not Asked: 389 (23.6%)	Not Asked: 82 (24.8%)	
	Not currently: 136 (6.9%)	Not currently: 114 (6.9%)	Not currently: 22 (6.6%)	
	Yes: 489 (24.7%)	Yes: 413 (25.0%)	Yes: 76 (23.0%)	
**DM**	0: 1,428 (72.1%)	0: 1209 (73.3%)	0: 219 (66.2%)	0.008
	1: 552 (27.9%)	1: 440 (26.7%)	1: 112 (33.8%)	
**Lung**	0: 1,867 (94.3%)	0: 1563 (94.8%)	0: 304 (91.8%)	0.035
	1: 113 (5.7%)	1: 86 (5.2%)	1: 27 (8.2%)	
**Heart**	0: 1,742 (88.0%)	0: 1462 (88.7%)	0: 280 (84.6%)	0.038
	1: 238 (12.0%)	1: 187 (11.3%)	1: 51 (15.4%)	
**Kidney**	0: 1,889 (95.4%)	0: 1577 (95.6%)	0: 312 (94.3%)	0.276
	1: 91 (4.6%)	1: 72 (4.4%)	1: 19 (5.7%)	
**Liver**	0: 1977 (99.8%)	0: 1647 (99.9%)	0: 330 (99.7%)	0.423
	1: 3 (0.2%)	1: 2 (0.1%)	1: 1 (0.3%)	

BMI: body mass index. Smoking: smoking. Alcohol: alcohol history. DM: history of diabetes mellitus. Lung: history of lung disease. Heart: history of heart disease. Kidney: history of kidney disease. Liver: history of Liver disease.

#### Performance of the model

**Fig 1:**
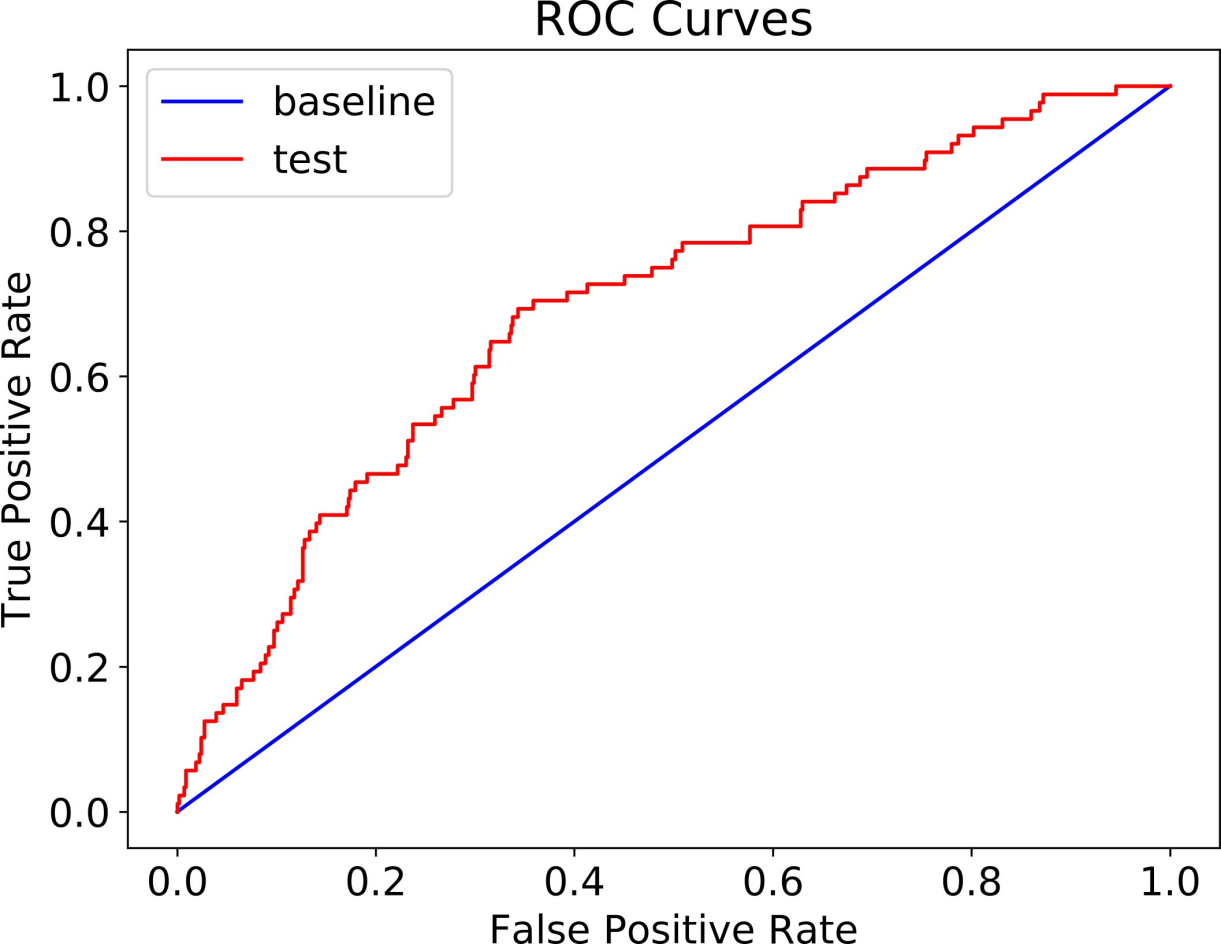
ROC curves mechanical ventilation prediction (AUC 68%).

The patient cohort of 1,306 patients (between 2/20/2020 and 4/17/2020) was used for training and validation of XGBoost model. After the model was trained and its hyperparameters were optimized in a k-fold cross-validation fashion, the performance of the model on a 20% randomly selected patients is summarized in Table 2. The accuracy of the model is 82.4% (95% CI: 0.047) with a negative predicative value (NPV) of 85.4% and specificity of 95.5%. The model was further tested on 674 COVID-19 patients, who visited ER departments between 4/18/2020 and 5/6/2020. The confusion matrix is shown in Table 3. The prediction accuracy is 86.2% (95% CI: 0.026) with a NPV of 87.8%, and specificity of 97.6%. AUC of ROC was 68% (Fig 1).

**Table 2:** Confusion matrix of prediction of mechanical ventilation in patients before 4/17 (accuracy: 82.4%).

	Non-MV	MV
Predicated	210	36
Non-MV		
Predicated	10	5
MV		

Non-MV: patients who were not mechanically vented. MV: patient who were mechanically vented. Predicted Non-MV: patients predicated to need mechanical ventilation. Predicated MV: patients predicated not to need mechanical ventilation.

**Table 3:** Confusion matrix of prediction of mechanical ventilation in patients between 4/17 and 5/5 (accuracy: 86.2%).

	Non-MV	MV
Predicated	572	79
Non-MV		
Predicated	14	9
MV		

Non-MV: patients who were not mechanically vented. MV: patient who were mechanically vented. Predicted Non-MV: patients predicated to need mechanical ventilation. Predicated MV: patients predicated not to need mechanical ventilation.

### Feature importance

**Fig 2:**
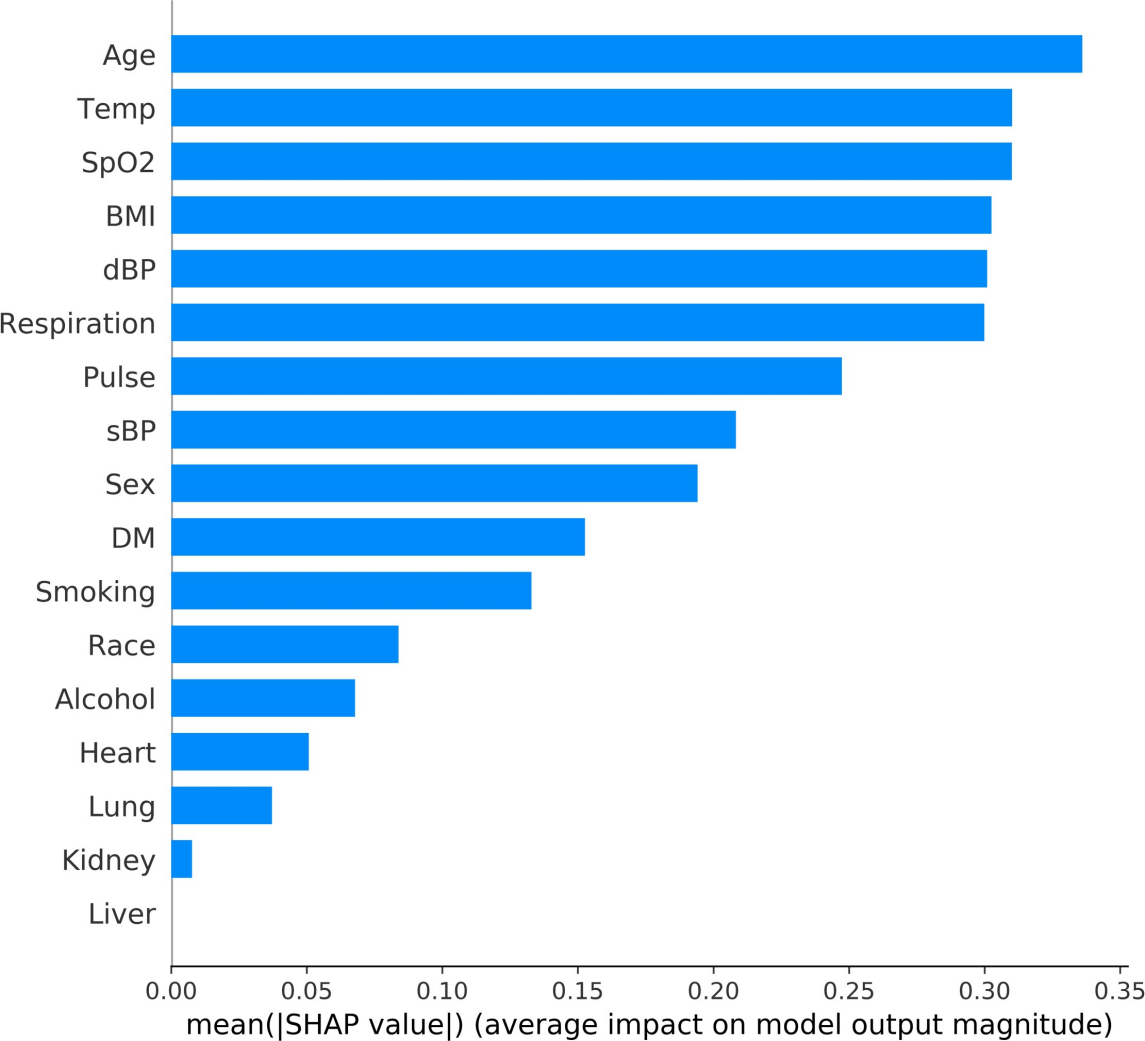
Feature Importance Ranking in mechanical ventilation prediction. This ranking measures impacts of features on prediction in descending order. Alcohol: alcohol history. BMI: body mass index. Smoking: smoking. dBP: first measurement of diastolic blood pressure at ER. DM: history of diabetes mellitus. Heart: history of heart disease. Kidney: history of kidney disease. Liver: history of Liver disease. Lung: history of lung disease. Respiration: first measurement of respiration rate at ER. Pulse: first measurement of pulse at ER. sBP: first measurement of systolic blood pressure at ER. Smoking: smoking history. SpO2: first measurement of blood oxygen saturation at ER. Temp: first measurement of temperature at ER.

**Fig 3:**
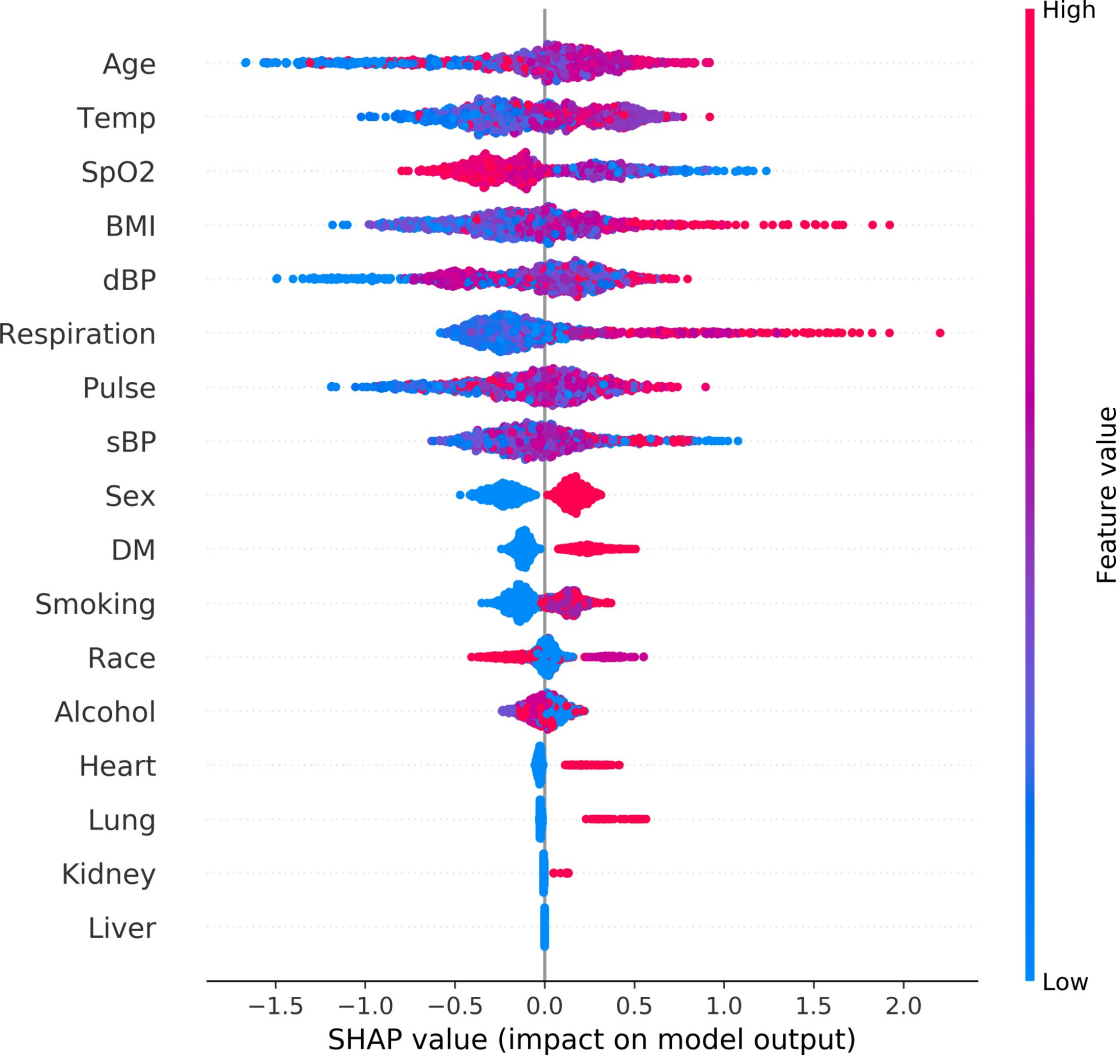
Global view of feature impact on mechanical ventilation prediction. Features are ranked in descending order of their accountability for the prediction. Each dot in the visualization represents one datapoint of a feature. Its color is related to the real data value: high value in red and low value in blue. The impact of each value is associated with higher or lower prediction, represented by SHAP values on x-axis. BMI: body mass index. Smoking: smoking. dBP: first measurement of diastolic blood pressure at ER. DM: history of diabetes mellitus. Heart: history of heart disease. Kidney: history of kidney disease. Liver: history of Liver disease. Lung: history of lung disease. Respiration: first measurement of respiration rate at ER. Pulse: first measurement of pulse at ER. sBP: first measurement of systolic blood pressure at ER. Smoking: smoking history. SpO2: first measurement of blood oxygen saturation at ER. Temp: first measurement of temperature at ER.

The features are ranked in descending order of their impact on prediction outcomes in Figs 2 and 3. Fig 2 shows the overall impact of the clinical features. In addition, contribution of each individual datapoint to prediction is demonstrated in Fig 3. Age was the most significant predictor of mechanical ventilation in COVID-19 patients. Increasing age was associated with a higher chance of MV. Patients with elevated temperature and an elevated RR had a higher chance of requiring MV. Likely, lower SpO2, history of DM and smoking were related to increased chance of MV.

### Prediction of mortality

This cohort included 3,491 COVID-19 patients, who visited ER departments of Beaumont Health and were subsequently hospitalized between 2/1/2020 and 5/4/2020 (Table 4). Their average age was 62.3 ± 17.5 years old and 51.4% were females. As with the MV cohort, the mortality cohort also had significant statistical differences among deceased and surviving patients in several categories including age, sex, race, BMI, smoking history, alcohol history, history of DM, history of lung disease, history of heart disease, and history of kidney disease (Table 4).

**Table 4:** Clinical characteristics of COVID-19 patients for mortality prediction.

	Total Patients (n = 3,491)	Survivors (n = 2,985)	Non Survivors (n = 506)	Significance
**Age**	62.3 ± 17.5	62.6 ± 17.5	74.2 ± 14.3	<0.001
**Sex**	Female 1,796 (51.4%)	Female 1,564 (52.4%)	Female 232 (45.8%)	0.006
	Male 1,695 (4.6%)	Male 1,421 (47.6%)	Male 274 (54.2%)	
**Race**	Asian: 69 (2.0%)	Asian: 62 (2.1%)	Asian: 7 (1.4%)	0.001
	African American: 1,823 (55.2%)	African American: 1,595 (53.4%)	African American: 228 (45.1%)	
	Caucasian: 1,415 (40.5%)	Caucasian: 1,166 (39.1%)	Caucasian: 249 (49.2%)	
	Other: 184 (5.3%)	Other: 162 (5.4%)	Other: 22 (4.4%)	
**BMI**	31.7 ± 8.8	31.8 ± 8.7	30.9 ± 9.2	0.023
**Smoking**	Never: 1664 (47.7%)	Never: 1463 (49.0%)	Never: 201 (39.7%)	<0.001
	Quit: 886 (25.4%)	Quit: 714 (23.9%)	Quit: 172 (34.0%)	
	Yes: 151 (4.3%)	Yes: 132 (4.4%)	Yes: 19 (3.8%)	
	Not Asked: 786 (22.5%)	Not Asked: 672 (22.5%)	Not Asked: 114 (22.5%):	
	Passive: 4 (0.1%)	Passive: 4 (0.1%)	Passive: 0 (0.0%)	
**Alcohol**	No: 1412 (40.4%)	No: 1178 (39.5%)	No: 234 (46.2%)	0.007
	Yes: 790 (22.6%)	Yes: 700 (23.5%)	Yes: 90 (17.8%)	
	Not Currently: 284 (8.1%)	Not Currently: 240 (8.0%)	Not Currently: 44 (8.7%)	
	Never: 158 (4.5%)	Never: 140 (4.7%)	Never: 18 (3.6%)	
	Not Asked: 847 (24.3%)	Not Asked: 727 (24.4%)	Not Asked: 120 (23.7%)	
**DM**	0: 2,483 (71.1%)	0: 2,144 (71.8%)	0: 339 (67.0%)	0.027
	1: 1,008 (28.9%)	1: 841 (28.2%)	1: 167 (33.0%)	
**Lung**	0: 3,309 (94.8%)	0: 2,840 (95.1%)	0: 469 (92.7%)	0.022
	1: 182 (5.2%)	1: 145 (4.9%)	1: 37 (7.3%)	
**Heart**	0: 3082 (88.3%)	0: 2,668 (89.4%)	0: 414 (81.8%)	<0.001
	1: 409 (11.75)	1: 317 (10.6%)	1: 92 (18.2%)	
**Kidney**	0: 3,329 (95.4%)	0: 2,858 (95.7%)	0: 471 (93.1%)	0.009
	1: 162 (4.6%)	1: 127 (4.3%)	1: 35 (6.9%)	
**Liver**	0: 3,484 (99.8%)	0: 2,980 (99.8%)	0: 504 (99.6%)	0.269
	1: 7 (0.2%)	1: 5 (0.2%)	1: 2 (0.4%)	

BMI: body mass index. Smoking: smoking. Alcohol: alcohol history. DM: history of diabetes mellitus. Lung: history of lung disease. Heart: history of heart disease. Kidney: history of kidney disease. Liver: history of Liver disease.

### Performance of the model

**Fig 4:**
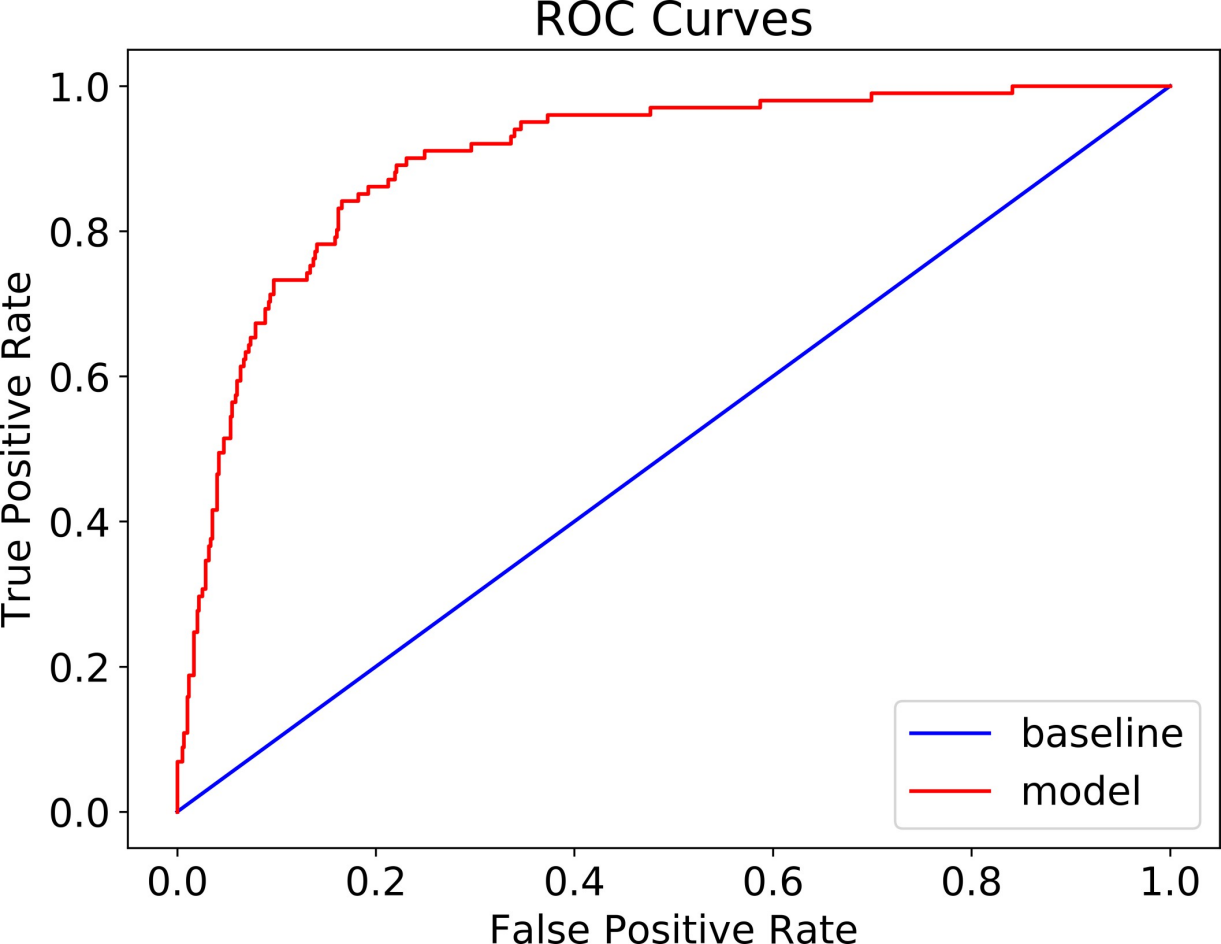
ROC curve of mortality prediction (AUC: 90%).

**Fig 5:**
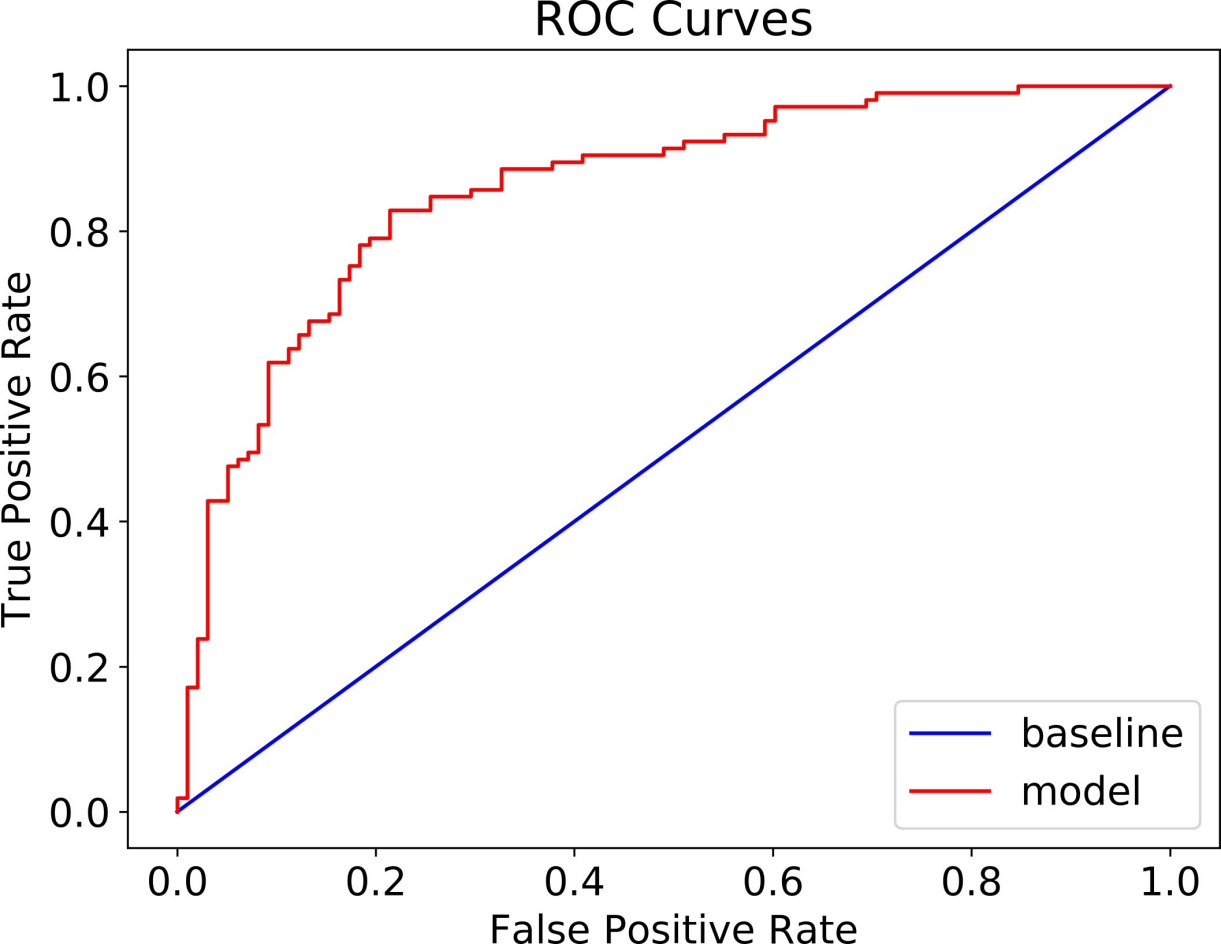
ROC of mortality prediction in balanced patient cohort (AUC: 86%).

The patient cohort was randomly split into training (80%) and testing (20%) groups to train and test CatBoost model. The confusion matrix is shown in Table 5. The accuracy of the model reached 88.3% (95% CI + 0.024) (Table 5) and the AUC of ROC is 90% (Fig 4). Because of the unbalanced nature of mortality in the COVID-19 patients, population of survived patients were randomly down-sampled to achieve a new balanced patient cohort, consisting of 506 survived patients and 506 deceased patients. The sample was then randomly split into training (80%) and testing (20%) to retrain CatBoost model. Its performance on testing group was shown in the confusion matrix in Table 6. The accuracy remained high at 80.3% (95% CI + 0.025). The NPV was 81.6%, and PPV was 79.0% with the balanced model. The AUC of ROC is 85% (see Fig 5).

**Table 5:** Confusion matrix for mortality prediction in COVID-19 patients (accuracy: 88.3%).

	Survived	Deceased
Predicated	574	43
survived		
Predicated	24	58
Deceased		

**Table 6:** Confusion matrix of prediction of mortality in COVID-19 patients (downsampled for balanced patient cohort) (accuracy: 80.3%).

	Survived	Deceased
Predicated	79	21
survived		
Predicated	19	84
Deceased		

### Feature importance

**Fig 6:**
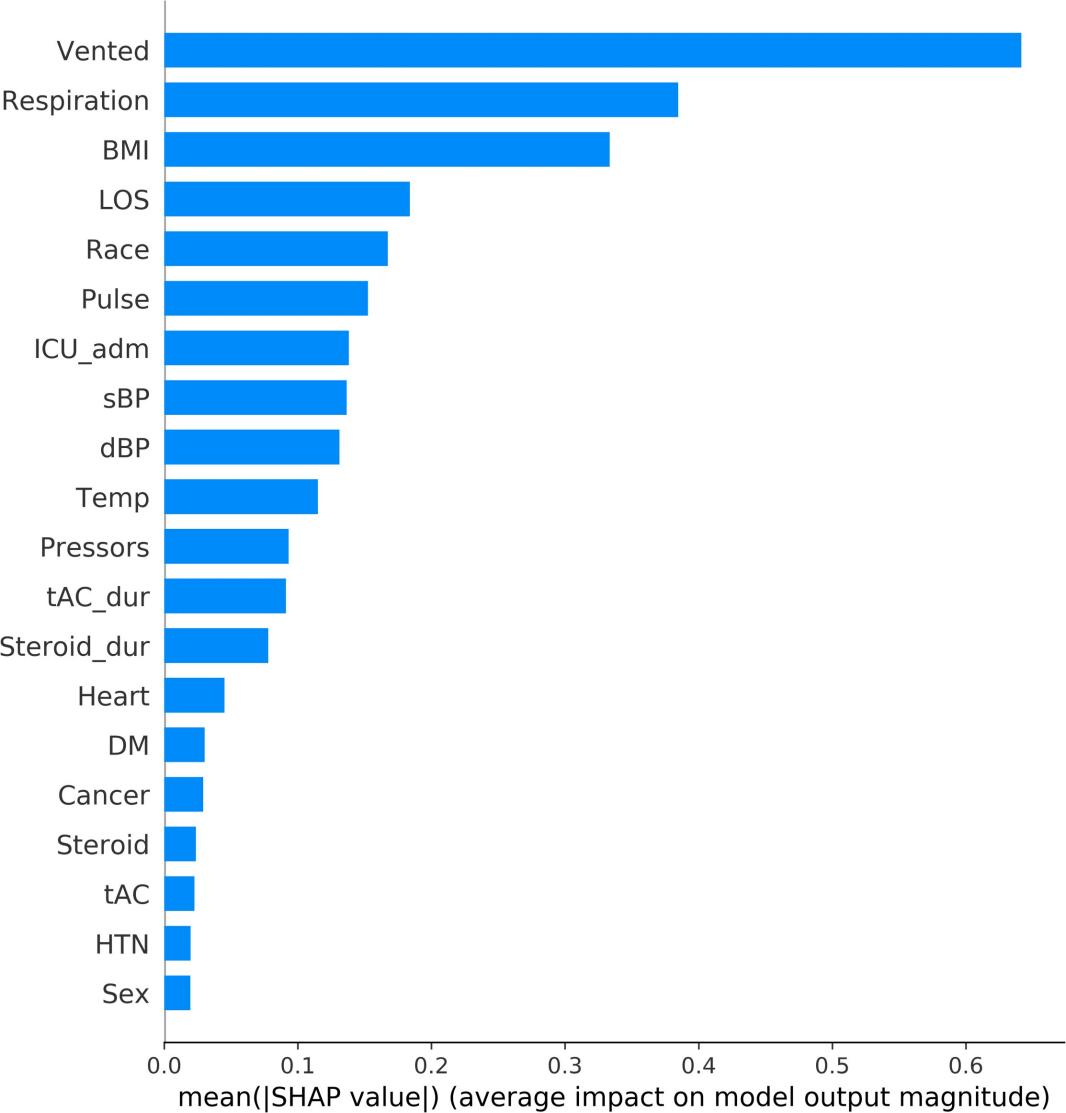
Feature importance ranking in mortality prediction. This ranking measures impacts of features on prediction in descending order. BMI: body mass index. Cancer: history of cancer. dBP: first measurement of diastolic blood pressure at ER. DM: history of diabetes mellitus. Heart: history of heart disease. HTN: history of hypertension. ICU_adm: whether a patient was admitted into ICU or not. LOS: length of stay in hospital. Pulse: first measurement of pulse at ER. Pressors: if a patient received vasopressor treatment. Respiration: first measurement of respiration rate at ER. sBP: first measurement of systolic blood pressure at ER. Steroid: whether a patient received steroid treatment. Steroid_dur: duration of steroid treatment. tAC: if a patient received anticoagulation treatment. tAC_dur: duration of anti-coagulation treatment. Temp: first measurement of temperature at ER. Vented: where a patient was mechanically vented or not.

**Fig 7:**
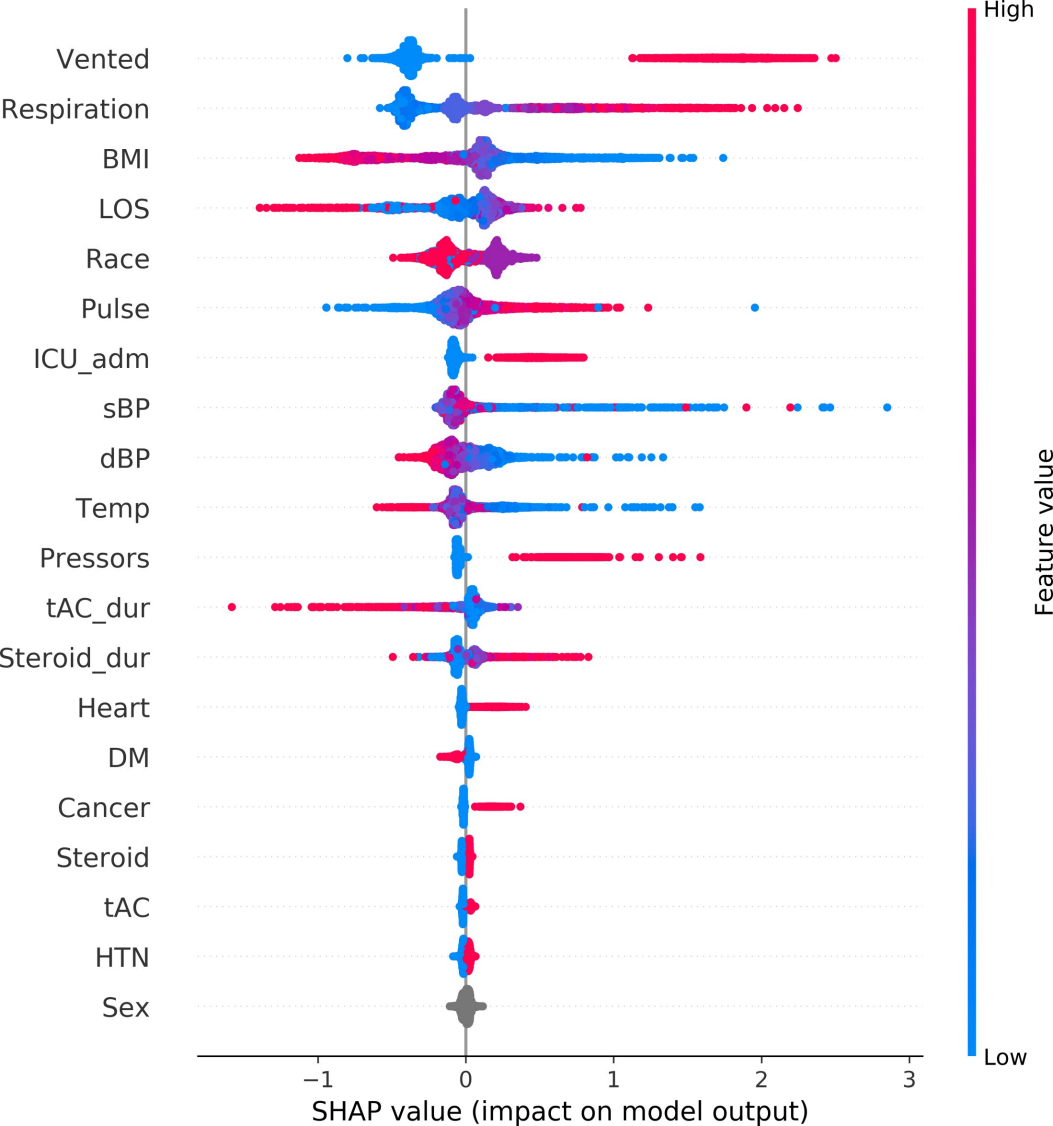
Global view of feature impact on mortality prediction. Features are ranked in descending order of their accountability for the prediction. Each dot in the visualization represents one datapoint of a feature. Its color is related to the real data value: high value in red and low value in blue. BMI: body mass index. Cancer: history of cancer. dBP: first measurement of diastolic blood pressure at ER. DM: history of diabetes mellitus. Heart: history of heart disease. HTN: history of hypertension. ICU_adm: whether a patient was admitted into ICU or not. LOS: length of stay in hospital. Pulse: first measurement of pulse at ER. Pressors: if a patient received vasopressor treatment. Respiration: first measurement of respiration rate at ER. sBP: first measurement of systolic blood pressure at ER. Steroid: whether a patient received steroid treatment. Steroid_dur: duration of steroid treatment. tAC: if a patient received anticoagulation treatment. tAC_dur: duration of anti-coagulation treatment. Temp: first measurement of temperature at ER. Vented: where a patient was mechanically vented or not.

The top-20 predictors using the CatBoost model are ranked in descending order by feature importance, as shown in Figs 6 and 7. Requirement of MV is the most important predictor of survival. Other important features included admission to the ICU, need for vasopressors, elevated respiration rate and pulse rate.

## Discussion

The highlight of this study is three-fold. First, we used a parsimonious ML algorithm to predict hard end points, such as mechanical ventilation with high specificity and NPV. The model is reliant on initial triage vitals in the ER, such as temperature and minimum oxygen saturation, and basic demographics such as age and BMI. Thus, it can assist physicians during the pandemic with making critical decisions of discharging home versus hospital admission. The model may also help with resource allocation and flow of operations for crisis management teams, especially with scarcity of ventilators. Secondly, the mortality prediction algorithm uses several key laboratory and other features in addition to patient characteristics and has consistent accuracy of over 85%. The major features were whether patient was receiving MV, had a high initial respiratory rate, longer length of stay and increased BMI. From a clinical standpoint, daily mortality assessment is crucial to determine the need for escalation of therapy, site of care decisions and goals of care discussion. The model may provide an avenue for dynamic deployment in the hospitals across the country to give “at a time risk of mortality” among admitted patients. Finally, ML algorithms used in model development are rigorous and can account for missing data and categorical nature of real-world data.

Arvind et.al. studied predictors of MV among 4087 patients from New York City, using random forest classifier, a supervised ML algorithm and demonstrated an AUC of 0.84, similar to our findings [14]. Unlike our model, they used 24-hour data to predict 72-hour risk of intubation in a time serious manner. But interestingly the highest weight in their model was elevated RR, again one of the major features in our initial predictive model. Compared to their study, we wanted to risk predict those at risk of intubation from the time of admission. Yan and colleagues from Wuhan, China predicted mortality from different biomarkers using ML and AI algorithms [15]. They had studied patients with COVID-19 from January 2020 to February 2020 even before the pandemic hit United States. They used XGBoost classifier as a predictor model. In their model high lactate dehydrogenase (LDH), low lymphocyte count (lymphopenia) and high levels of high sensitivity C-reactive protein (hs-CRP) were found to be predictors of mortality. Their model has high level of accuracy (90%) in predicting mortality 10 days in advance but their sample size was small (n = 485). Compared to our study, Yan only studied biomarkers, while we included all parameters including demographics, vitals, comorbidities, and other variables like need for MV and need for vasopressors. Therefore, biomarkers were not ranked high in our prediction model. In another study, Wu et.al, studied COVID 19 patients from China, as well as, other countries like Italy and Belgium to train and validate a clinical prediction model for severity of pneumonia [16]. Non-severe patients were treated at home or mobile hospitals, while severe patients required higher level of care including ICU care. They did not use machine learning instead they used clinical scoring system with all available demographics, comorbidities and investigational data. During validation of model they achieved high accuracy with AUC ranging from 0.84 to 0.89. Age was one of the important predictors. Similar to their results our machine leaning algorithms found similar significance for age in mortality prediction.

Cheng and colleagues used a random forest model to predict ICU admission within 24 hours among 1987, COVID-19 patients admitted to non-ICU units of a large hospital system in NY [17]. Their model had good specificity of 76.3% with an accuracy of 76.2% (95% CI: 74.6–77.7%). However, the population included majority of women and patients younger than 65. They used 9639 feature vectors with data from each day of non-ICU hospital stay. The final model in the study found strongest predictors of ICU admission to be respiratory rate and white blood cell count. Other features included markers of respiratory failure, systemic inflammation, shock, and renal failure. Paradoxically patients older than 65 years had lower ICU transfer rate despite high mortality. Compared to Cheng’s study, our first algorithm predicted the need for MV during the pandemic. The boundaries for ICU were not clear during the surge in our hospital system with many patients admitted to progressive care units, which were equipped and staffed to function as ICU. Further, we included all ER patients at the time of evaluation and not admitted patients. Our aim was to develop a model using minimal features to practically predict, who is not at risk of MV to determine who may be safely discharged.

Yadaw and associates examined mortality predictors in a large cohort of 5,051 COVID-19 patients using XGBoost similar to our study [18]. Of the initial 20 features that were selected the consistent features in a SHAP model that showed reliable mortality prediction were minimum oxygen saturation, age, type of encounter, maximum body temperature, and use of hydroxychloroquine during treatment. Their model performed similarly to ours with an AUC of > 0.9. The findings in the Yadaw study are similar to ours and reiterates the robustness of ensemble-based ML classification algorithm. Although, we used XGBoost for MV prediction, we felt CatBoost was a superior technique for mortality prediction in its ability to handle categorical and numerical data.

We acknowledge out study had some limitations. Although, we had a large number of patients with several important features, our dataset for MV and mortality were unbalanced affecting the sensitivity of the results. Since our goal was to have a high accuracy and NPV in the ER, we kept the same sample. We did down sample the mortality data to balance the group with similar accuracy. We used admission vitals instead of time series data. Consistent with our decision on using admission vitals for disease stratification, Fernandes and colleagues used ML and natural language processing algorithm to predict ICU admission among patients presenting to ER [19]. As with our results in COVID-19 patients, they noted initial vitals including heart rate, oxygen saturation, RR and sBP to be highly correlated to ICU admission. We also did not use time-series data for mortality prognostication for patients in the ICU, although length of stay was one of the major predictive features for mortality in our model. In future studies, we will further strengthen the model by using time-series data.

Lastly, one very important point we want to highlight is contextual factors. Overall mortality in mortality prediction model was around 14%. At the time of this study state of Michigan had significant number of cases of COVID 19 compared to other states in United States. Science about COVID-19 is still not clearly understood but during early part of pandemic understanding about the disease was negligible. Crisis, fear amongst healthcare workers, lack of resources and lack of understanding might have contributed to higher need for mechanical ventilation and/or mortality. These contextual factors are very important while comparing data from one study to other. It is not possible to conclude meaningfully without putting these global factors in to consideration which might be limitation of our study as well as limitation of most of the studies we have cited.

## Conclusion

Machine learning models using XGBoost for need for MV and catBoost for prediction of mortality amongst COVID-19 patients are accurate with high specificity and NPV. Simple factors like age and vitals can predict need for mechanical ventilation, thus helping ER physicians to decide the need for admission to hospital versus discharging patient home. Patients requiring mechanical ventilation, higher respiratory rate and BMI were amongst the top predictors for mortality.
